# Presence and Maturation Dynamics of Mandibular Third Molars and Their Influence on Late Mandibular Incisor Crowding: A Longitudinal Study

**DOI:** 10.3390/ijerph181910070

**Published:** 2021-09-25

**Authors:** Martina Zigante, Andrej Pavlic, Luka Morelato, Vaska Vandevska-Radunovic, Stjepan Spalj

**Affiliations:** 1 Department of Orthodontics, Faculty of Dental Medicine, University of Rijeka, Kresimirova 40, 51000 Rijeka, Croatia; pavlic.andrej@yahoo.com (A.P.); stjepan.spalj@fdmri.uniri.hr (S.S.); 2 Department of Pediatric Dentistry, Faculty of Dental Medicine, University of Rijeka, Kresimirova 40, 51000 Rijeka, Croatia; 3 Department of Oral Surgery, Faculty of Dental Medicine, University of Rijeka, Kresimirova 40, 51000 Rijeka, Croatia; morelatoluka@gmail.com; 4 Department of Orthodontics, Institute of Clinical Dentistry, University of Oslo, Geitmyrsveien 71, 0317 Oslo, Norway; vaska.vandevska-radunovich@odont.uio.no; 5 Department of Dental Medicine, Faculty of Dental Medicine and Health, Josip Juraj Strossmayer University of Osijek, Crkvena 21, 31000 Osijek, Croatia

**Keywords:** Cameriere’s index, crowding, growing subjects, Little’s irregularity index, malocclusions, third molars

## Abstract

The objective of this study was to investigate the relationship between the absence, presence and dynamics of mandibular third molar development and the occurrence and amount of late mandibular incisor crowding. Dental plaster casts and panoramic radiographs of 72 orthodontically untreated subjects from the Nittedal growth study, Norway were analyzed. The subjects were recalled for a checkup at 12, 15, 18 and 21 years of age. Mandibular incisor crowding was assessed using Little’s irregularity index and dental maturation of the third molars by the Cameriere’s index. The majority of the subjects (64%) had ≥1 mm increase in irregularity; 22% experienced an increase of 0.1–0.9 mm and 14% had unchanged or decreased irregularity. Incisor irregularity increased with age, regardless of absence or presence of third molars. The amount of change in incisor irregularity from 12 to 21 years did not differ significantly between subjects with hypodontia of third molars, extraction and those with third molars present. No differences were observed between erupted, unerupted or impacted third molars. No correlation was found between the amount of change in irregularity and maturation of the third molars. In conclusion, occurrence and amount of mandibular late incisor crowding is not significantly influenced by the presence of mandibular third molars or their development dynamics.

## 1. Introduction

The relationship between third molars and mandibular incisor crowding is one of the most debated and studied fields in orthodontics, and despite all, is still quite controversial. Late crowding is considered to have multifactorial etiology, whereas etiological factors may differ between individuals. Proposed etiological factors include differential growth of the jaws, functional and parafunctional pressure of the soft tissues, muscular imbalance and distribution of the anterior component of the occlusal force [1,2]. Many studies have attempted to clarify and evaluate the third molars and incisor crowding interrelationship [3,4,5,6,7,8]. They focused on third molar angulations, position, space, or their extraction, but none analyzed their maturation dynamics. Mandibular incisor crowding is highly prevalent, as up to 40% of the general population has moderate to severe crowding [9]. Given the esthetic demands, maxillary incisor crowding is one of the most frequent reasons for seeking orthodontic treatment [10]. However, with aging, there is a gradual decrease of exposure of upper incisor, accompanied by an increase in lower incisor exposure [11], making the lower crowding more visible therefore compromising smile esthetics.

Skeletal maturation can be influenced by environmental and hereditary factors. On the other hand, teeth are much more reliable for age estimation because their tissues do not undergo continuous remodeling processes. Over time, versatile dental age estimation methods have been developed. In 2006, Cameriere [12] presented a method based on a measurement of open apices of the teeth. The method was originally applied to Italians and then applied to different European and non-European subjects [13,14,15,16]. It was reported as very accurate, because it showed that variability between samples did not significantly influence the regression formula [17]. When applied to third molars, this method showed a very high percentage of correctly classified cases in European subjects [18].

Crowding as an occlusal trait becomes more common during dentition development and aging, due to maturational, regressive and degenerative factors [1,2,19]. Incisor crowding most commonly manifests as tooth rotations and labiolingual displacement from the arch line, often followed by different amounts of mesiodistal overleap of contact points [20]. Late crowding is observed in mandibular incisors during late adolescence, and it is considered to be a late expression of primary crowding [1,19].

An increase in mandibular incisor crowding was reported to occur between 13 and 26 years, in late adolescence and early adulthood [21,22]. It often coincides with the eruption of mandibular third molars, which might imply their causal role. However, it seems that the role of mandibular third molars in anterior crowding cannot be categorically denied [23].

Late mandibular incisor crowding is observed in both treated and untreated subjects, and worsens with age, most evidently due to a decrease in arch length and perimeter and mandibular dental arch becoming more square-shaped [21,24,25,26].

Many literature reviews [27,28,29] have attempted to affirm the relationship between third molars and crowding, however, due to questionable methods, lack of standardization, various inclusion criteria and study designs, definite conclusion on this interrelationship cannot be set [29]. Some authors attributed incisor crowding to the mesial pressure exerted by the mandibular third molar [4], on the other hand, others do not consider this pressure capable of causing anterior crowding [3]. Some research findings report that no strong relationship exists between the third molar eruption level, space, and angulations to mandibular anterior crowding [6]. It seems that the only connection between crowding and eruption of the third molars is the concurrent occurrence of the two phenomena [30].

This study aimed to investigate absence, presence and mandibular third molar development dynamics, i.e., rate; occurrence and amount of late mandibular incisor crowding and their possible interrelationship.

We hypothesized that decelerated development and absence of eruption of mandibular third molars could influence the amount of mandibular incisor crowding.

## 2. Materials and Methods

The sample of the study was part of the Nittedal growth study, collected by the Department of Orthodontics, University of Oslo, Norway. It includes documentation of 4229 orthodontically untreated subjects who were residents of Nittedal County in Norway born between 1958 and 1972. Subjects had no significant malocclusions nor facial disharmonies at the start of follow-up at six years of age and received no orthodontic treatment. They were followed up every three years from the ages of 6 to 21 years. However, the number of subjects with complete documentation for a period of 12 years was limited since those that had orthodontic treatment were excluded from the sample.

Inclusion criteria were presence of panoramic radiographs and plaster casts at the ages of 12, 15, 18 and 21. After selection of the cases with required documentation, the sample consisted of 72 subjects (47% female) longitudinally followed from 12 to 21 years, every 3 years.

**Figure 1:**
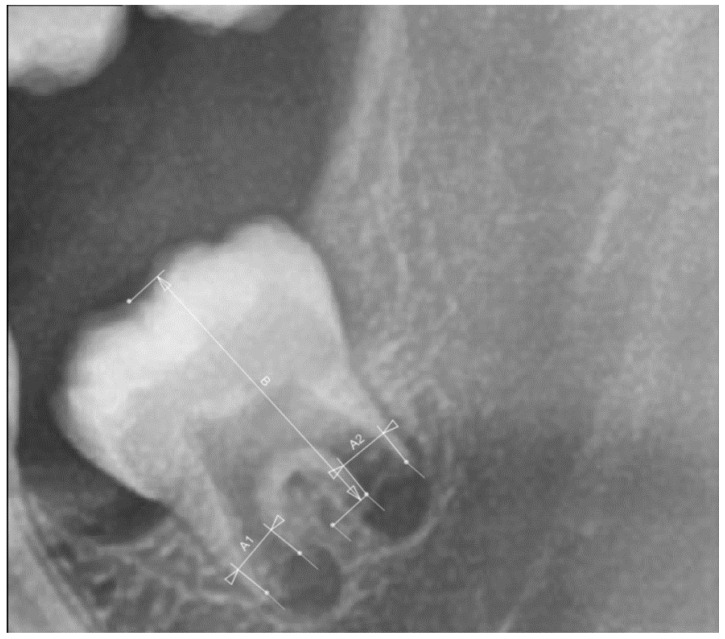
Cameriere’s method measurement in AudaxCeph software.

The maturation stage of the left mandibular third molar was determined on panoramic radiographs at the ages of 15, 18 and 21 using the Cameriere’s method [12]. The maturation stage was not evaluated at the age of 12 since mandibular third molars were insufficiently developed for this analysis. Cameriere’s method was performed by measuring the distances between inner walls of open root apices of mesial (A1) and distal (A2) roots which were then summed up and then divided by measured tooth length (B) [12] (Figure 1). This way, as the author stated differences in magnification and angulation among the different x-rays is controlled for [12]. Congruence of maturation of left and right mandibular third molars was evaluated in 30 subjects at every age.

Late incisor crowding was measured on plaster casts using Little’s irregularity index (the irregularity index) [20] which quantifies the aberration of contact points of mandibular incisors as a sum of displacement of contact points of neighboring teeth from canine to canine.

**Figure 2:**
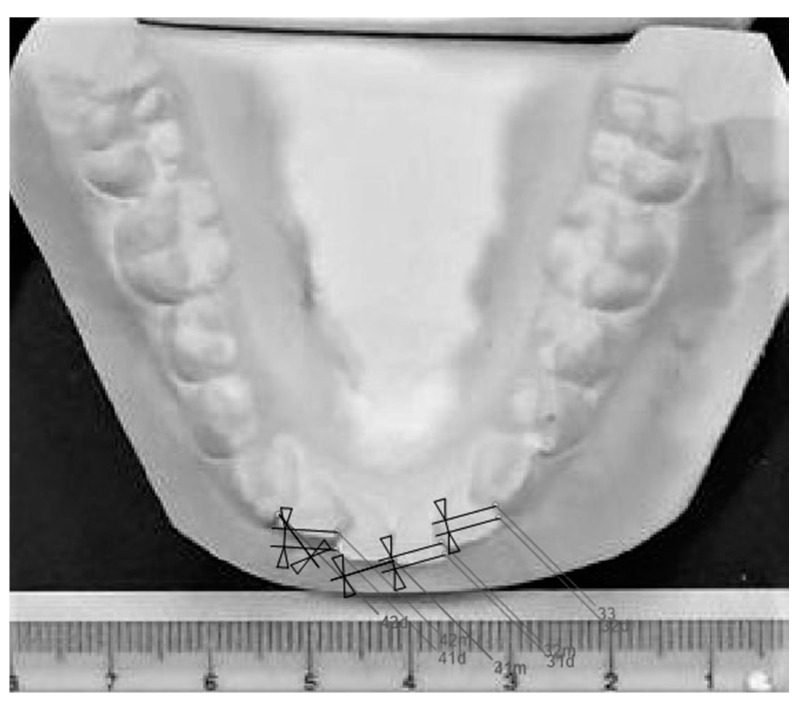
Little’s irregularity index measurement in AudaxCeph software.

Subjects’ plaster casts were photographed on a surface containing a gauge, from a normed distance of 0.6 m, to calibrate and facilitate their analysis in software AudaxCeph (Audax, Ljubljana, Slovenia) (Figure 2). One researcher (M.Z.) measured irregularity index and the other one (L.M.) measured the rate of third molar maturation to control observer bias. Repeatability analysis for Little’s irregularity index and Cameriere’s measurements (third molar maturation) was performed on 30 cases in the two-week interval by two raters. Intra- and inter-rater agreement were assessed for both measurements.

Statistical analyses included a t-test, Pearson and linear and logistic regression. The intraclass correlation coefficient (ICC) was used for the analyses of intra- and inter-rater repeatability, and concordance between left and right third molars and the following interpretation was used: r < 0.50 = poor, 0.50–0.75 = fair, 0.75–0.90 = good, >0.90 = excellent. All statistical analyzes were made in commercial software IBM SPSS 22 (IBM Corp, Armonk, NY, USA).

## 3. Results

Excellent intra- and inter-rater agreement for Cameriere’s and Little’s indices were present (intraclass correlation coefficient in range 0.995–0.999) for two raters, MZ and LM.

Descriptive statistics of the sample is presented in Table 1.

**Figure 3:**
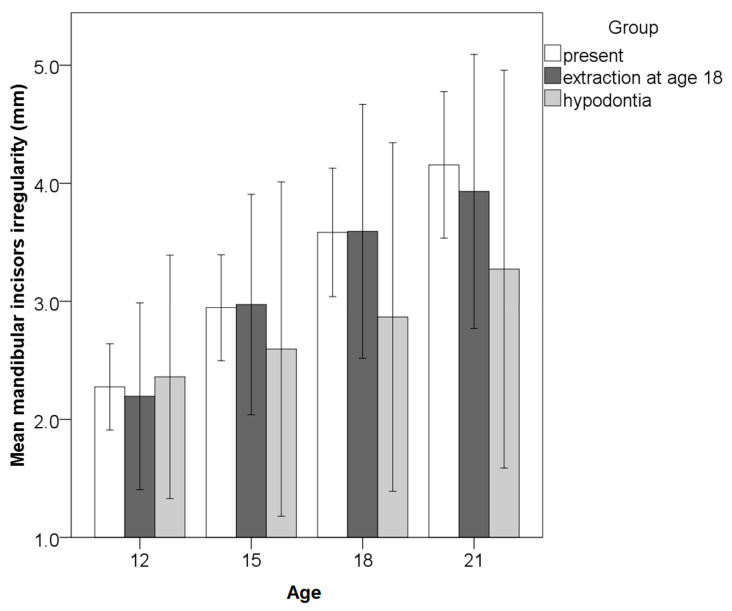
Comparison of mandibular incisor irregularity (The irregularity index) from the ages of 12 to 21 years between the groups with present, hypodontia or extracted third molars (whiskers present 95% confidence intervals).

**Figure 4:**
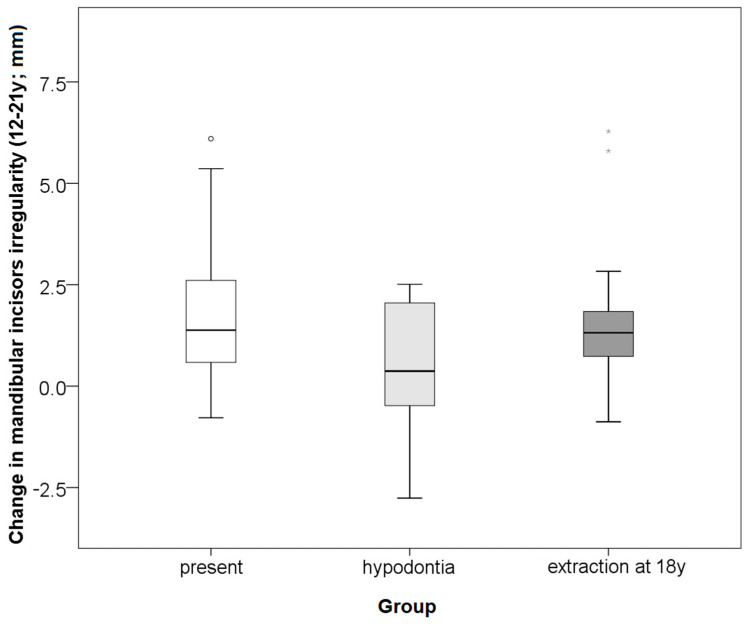
Comparison of change in mandibular incisor irregularity (The irregularity index) from 12 to 21 years between the groups with present, absent and extracted third molars. Circles and asterisk present outliers and extremes.

**Figure 5:**
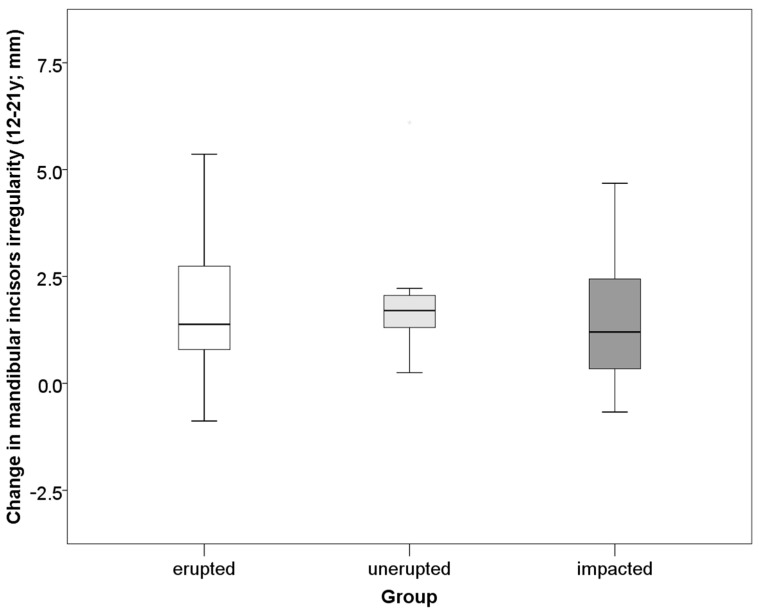
Comparison of change in mandibular incisor irregularity (The irregularity index) from 12 to 21 years between the groups with erupted, unerupted and impacted third molars.

The change in mandibular incisor irregularity from 12 to 21 years of age was in the range −2.8–6.3 mm (mean 1.6 ± 1.7). The majority of the subjects (64%) had ≥1 mm an increase in irregularity; 22% experienced increase of 0.1–0.9 mm and 14% had unchanged or decreased irregularity. An increase was more frequent between 12–15 years (in 86.2% of cases). Additional crowding was observed between 15 and 18 years in 84.6% and between 18 and 21 years of age in 86.6% of cases. Hypodontia of mandibular third molars was present in 11% of the subjects (in half unilateral), while 22% had their third molars removed at the age of 18 (57% unilateral). Among the subjects with present mandibular third molars at the age of 21, 45% had erupted (33% bilateral, 11% unilateral), 22% unerupted (8% bilateral and 14% unilateral), 33% impacted (27% bilateral and 6% unilateral). Incisor irregularity increased with age, regardless of the absence or presence of third molars (Figure 3). The amount of change in incisor irregularity from 12 to 21 years and incidence of additional irregularity did not differ significantly between subjects with hypodontia of third molars, extraction and those with third molars present (Figure 4, Table 2). Both incidence and increase of irregularity were the lowest in cases with hypodontia. Neither incidence nor increase of incisor irregularity differed significantly between the subjects with erupted, unerupted and impacted mandibular third molars (Figure 5, Table 2). The results were similar when analyzed separately for unilateral or bilateral extractions, hypodontia, eruption and/or impaction. Therefore, the results are pooled (Figure 3, Figure 4 and Figure 5, Table 2). Hypodontia of at least one mandibular third molar was taken as a dominant condition, followed by extraction of at least one mandibular third molar. If erupted, impaction was taken as dominant condition, followed by non-eruption of at least one mandibular third molar. The changes in irregularity were similar from 18 to 21 years in those that extracted and retained their third molars (0.6 ± 0.6 vs. 0.4 ± 0.9). The incidence of additional crowding after the age of 18 was similar in extraction and non-extraction cases (48 vs. 40%). Differences in the number of changes between males and females were not statistically significant.

Agreement in the maturation of left and right mandibular third molars was good to excellent, ranging from 0.863–0.963 and it was lowest at the age of 15. The rate of the third molar apex closure in the whole sample was bigger from the ages of 15 to 18, than from the ages of 18 to 21 with a large effect size (*p* < 0.001; r = 0.885; Table 1). Maturation of the third molar from the ages of 18 to 21 was more pronounced in females than males with a large effect size (0.31 ± 0.17 vs. 0.16 ± 0.10, *p* < 0.001; r = 0.503), while somewhat higher in males between 15–18 years in comparison to females (0.68 ± 0.22 vs. 0.62 ± 0.24).

The amount of change of lower incisor irregularity between the ages of 12 and 21 was not linearly correlated with the rate of third molar apex maturation at any age range (15–18 years, 18–21 years nor 15–21 years). When the influence of sex was controlled for in the linear regression, likewise, there was no correlation between the amount of change of lower incisors and maturation of third molars. The presence of mandibular third molars, their developmental rate and sex were not significant predictors of the occurrence of mandibular incisor irregularity in logistic regression models.

## 4. Discussion

The present study confirmed that mandibular third molars are not related to late incisor crowding in the mandible. The hypotheses that decelerated development and absence of eruption of third molars could influence the amount of incisor crowding are rejected.

Our study found that the hypodontia of mandibular third molars was present in 11% of the subjects, which is less often than in Malaysian-Chinese orthodontically untreated subjects whereas the overall hypodontia of the third molars was 30–33% [31]. However, the authors evaluated both the maxilla and the mandible, and the hypodontia of the third molars was found less often in the mandible than in the maxilla [31].

Hypodontia of third molars in the mandible did not reduce the odds for late incisor crowding and subjects with hypodontia had similar amounts of incisor crowding as those with third molars present. Our findings support previous studies which showed that neither presence nor absence of mandibular third molars influence lower incisor crowding [24,32]. However, it seems that agenesis of third molars could be associated with microdontia of other teeth, while the presence of the third molars is more often associated with impacted canine. [33].

This study has shown that extraction of third molars at the age of 18 did not reduce the amount of change in the irregularity of mandibular incisor or odds for its occurrence in the next three years, which is similar to what has been previously reported [3]. Unilateral extraction of mandibular third molars may slightly reduce crowding on the extraction side [4], but with questionable clinical relevance (−0.4 to +0.8 mm), hence extraction is not justified [3]. The present study confirmed that impaction of mandibular molars is not related to incisor crowding which agrees with previous findings, including analyzed depth and angulation [5].

One study identified the relationship between mandibular crowding and the angulation of mandibular third molars, suggesting that calculation of the Ganss ratio could serve as an indication for removal of third molars [7]. However, this study was recently discredited due to the restricted sample size [8]. Impaction of the mandibular third molar seems to be associated with increased cranial width and decreased cranial facial height [34].

It is evident from the present research that people without third molars also exhibit an increase in incisor irregularity with age, although in a slightly smaller amount and somewhat less frequently. However, there is a large interindividual variability and given the small number of people with hypodontia in the sample and lack of statistical significance, it cannot be argued that there is a relationship. It is questionable whether it would be justified to perform a germectomy of the third molars at an early age just to slow down the occurrence of crowding.

Even though some studies suggest that crowding differs between the erupted and extracted or absent third molars group [35], according to our study, it seems that neither impaction nor eruption of third molars influences the amount of crowding. Crowding is also similar regardless of unilateral or bilateral extraction, hypodontia, eruption, and/or impaction of third molars. Also, due to ethical considerations, all the studies of the untreated subjects are based on historical samples which have not experienced secular growth trends over the past century, hence making their conclusions of questionable quality. Studies include opposing reports of whether secular trends influence dental maturity or not [36,37]. Some research implies that a positive secular trend in dental maturity, i.e., faster dental development, is observed [37]. Turkish researchers report that more rapid dental maturation is observed in girls in comparison with boys of the same age, even though no significant generational secular trends were observed [36].

According to the present study, the maturation rate of third molars does not influence the late crowding of mandibular incisors. To our knowledge, there were no studies investigating the relationship between third molar maturation and dental crowding. However, one study investigated the synchronism of dental maturation and facial development and its impact on crowding. They concluded that asynchronous dentofacial development could partially explain the frequency of dental crowding in modern populations [38]. It also seems that a secular trend is observed in the development of third molars: the onset begins earlier and then decelerates [39]. Calcification and eruption of third molars are reported to be affected by ethnicity [40]. It could be that secular trends in the development of the dentofacial complex, transition to an agricultural lifestyle and reduced masticatory function [41,42] are behind earlier third molar maturation and incisor crowding.

The study’s limit is its small sample size, but it is dependent on the available data of subjects with complete documentation from the historical growth study. Another limitation is the exclusion of subjects from the historical growth study due to orthodontic treatment. Based on the data obtained in the present research for detection of statistically significant differences in the increase of Little’s irregularity from 12 to 21 years between persons with hypodontia and with wisdom teeth present (mean difference 1.3 and standard deviation 1.8), the study would require a sample size of 31 for each group (present and absent third molars), to achieve a power of 80% and a level of significance of 0.05. The sample size was sufficient in the group with wisdom teeth present, but not in the group with absent wisdom teeth. The minimum required sample size for multiple regression analysis of the influence of two predictors (wisdom tooth maturation developmental rate from 15 to 21 years and sex) on the change of irregularities, with a medium effect size f^2^ = 0.073 (calculated from R^2^ = 0.068 obtained from this study), power of 80% and a significance level of 0.05, would be 134 subjects with wisdom teeth.

The strength of this study is the longitudinal research design that followed the occurrence and amount of the mandibular incisor crowding during the time, as well as the rate of maturation and position of the mandibular third molars. Therefore, several biases were controlled for, namely, selection bias and temporal bias, in comparison to retrospective and cross-sectional study designs. Since one investigator measured the irregularity index and the other investigator measured the rate of third molar maturation, observer bias was also controlled for. Another strength of this study is the multivariable regression analysis which controlled for more than one confounder at a time and allowed interpretation of each confounder individually.

## 5. Conclusions

The occurrence and amount of mandibular late incisor crowding are not significantly influenced by the presence of mandibular third molars or their development dynamics.

## Figures and Tables

**Table 1:** Descriptive statistics of the sample.

Variable	Mean ± SD	Median (IQR)	Range (Min–Max)
Irregularity index 12 years (mm)	2.4 ± 1.3	2.2 (1.4–3.2)	0.4–5.7
Irregularity index 21 years (mm)	3.9 ± 2.0	3.8 (2.3–4.7)	0.9–10.4
Change of irregularity index 21–12 years (mm)	1.6 ± 1.7	1.4 (0.5–2.5)	−2.8–6.3
Cameriere’s index 15 years	0.95 ± 0.37	0.89 (0.77–1.10)	0.28–2.27
Cameriere’s index 18 years	0.28 ± 0.24	0.20 (0.10–0.39)	0.01–0.97
Cameriere’s index 21 years	0.05 ± 0.11	0.01 (0.01–0.02)	0.00–0.58
Change of Cameriere’s index 18-15 years	0.68 ± 0.23	0.67 (0.50–0.82)	0.24–1.36
Change of Cameriere’s index 21-18 years	0.23 ± 0.15	0.20 (0.11–0.34)	0.01–0.68
Change of Cameriere’s index 21-15 years	0.90 ± 0.31	0.87 (0.74–1.06)	0.26–2.00

**Table 2:** Comparison of the change of the Little’s irregularity index in the period 12–21 years between the groups with present, hypodontia and extracted third molars.

Group	Incidence of Additional Irregularity (≥1 mm)	Incidence of Additional Irregularity (≥2 mm)	Mean ± SD	Median (IQR)	Range (Min–Max)
Third molars present at 21 years	65%	38%	1.8 ± 1.6	1.4 (0.6–2.7)	−0.8–6.1
Hypodontia of third molars	38%	25%	0.5 ± 1.8	0.4 (−0.6–2.2)	−2.8–2.5
Extraction of the third molars at 18 years	75%	19%	1.7 ± 2.0	1.3 (0.6–1.9)	−0.9–6.3
Impacted at 21 years	53%	29%	1.3 ± 1.4	1.2 (0.3–2.5)	−0.7–4.7
Unerupted at 21 years	82%	27%	1.9 ± 1.5	1.7 (1.3–2.2)	0.3–6.1
Erupted at 21 years	65%	39%	1.7 ± 1.6	1.4 (0.7–2.8)	−0.9–5.4

## Data Availability

The data presented in this study are available on request from the corresponding author if data sharing is approved by ethics committee. The data are not publicly available due to data protection laws and conditions stated by the ethics committee.
